# *TNFRSF13B* Diversification Fueled by B Cell Responses to Environmental Challenges—A Hypothesis

**DOI:** 10.3389/fimmu.2021.634544

**Published:** 2021-02-17

**Authors:** Marilia Cascalho, Jeffrey L. Platt

**Affiliations:** Department of Surgery and Department of Microbiology and Immunology, University of Michigan, Ann Arbor, MI, United States

**Keywords:** B-lymphocyte, *TNFRSF13B*, antibodies, T cell-dependent antibody response, T cell-independent antibody responses

## Abstract

B cell differentiation and memory are controlled by the transmembrane activator and CAML interactor (TACI), a receptor encoded by *TNFRSF13B*. *TNFRSF13B* mutations are frequently found in common variable immunodeficiency (CVID) and in IgA -deficiency; yet, ~98% of those with mutant *TNFRSF13B* are healthy. Indeed, *TNFRSF13B* is among the 5% most polymorphic genes in man. Other mammals evidence polymorphism at comparable loci. We hypothesize that *TNFRSF13B* diversity might promote rather than detract from well-being by controlling key elements of innate immunity. We shall discuss how extraordinary diversity of *TNFRSF13B* could have evolved and persisted across diverse species of mammals by controlling innate and adaptive B cell responses in apparently paradoxical ways.

## Introduction

B cell responses are often characterized as T cell-independent or T cell-dependent that differ on how T cells are engaged. In “T-independent B cell responses” antigens with repetitive epitopes, such as polysaccharides, engage B cell antigen receptors and/or toll-like receptors and in doing so induce proliferation and plasma cell differentiation. The antibodies produced in T cell-independent responses may appear relatively soon after introduction of antigen, but not immediately as in recall responses. The Ig variable region genes encoding these antibodies typically lack extensive somatic mutation (1). Repeated exposure to antigen that had generated a T cell-independent response does not hasten and amplify the response [i.e., B cell memory is not manifest; (1)]. In contrast, the T-dependent pathway requires T cell help and is associated with responses to protein antigens. Proteins are processed and peptides presented on major histocompatibility class II molecules expressed by antigen presenting cells that activate cognate CD4-positive T cells. These T cells in turn, engage cognate B cells activated by the same antigen by binding peptide MHC class-II complexes and co-receptors such as CD40. T cell-dependent responses induce long-lived memory responses and are associated with high affinity binding antibodies produced by plasma cells descending from the germinal centers in the secondary lymphoid organs (1). Naturally occurring polymorphisms in the *TNFRSF13B* gene differentially control T-dependent and T-independent pathways of antibody production.

## *TNFRSF13B* and the Control of Antibody Production

*TNFRSF13B* encodes the tumor necrosis factor superfamily member 13B, a transmembrane receptor of lymphocytes that recognizes a proliferation induced ligand (APRIL) and B cell activation factor (BAFF), members of the tumor necrosis ligand family (2). TNFRSF13B also binds heparan sulfate chains associated with syndecan-2 and−4 cores (3). The signaling events initiated by TNFRSF13B are complex and intersect with signaling by Toll-Like receptors (TLRs) and will be only briefly summarized here. Binding of BAFF and APRIL to the cysteine rich domain of the receptor closest to the cell membrane (CRD2) engages TNFR—associated factors (TRAF 2, 5, and 6) and activates NF-kB, c-Jun NH2-terminal kinase (4) and activator protein 1 (AP-1) (5). TNFRSF13B interacts with calcium modulator and cyclophilin ligand (CAML), which in turn activates calcineurin and nuclear factor of activated T cells (NFAT) (6). TNFRSF13B is sometimes called “transmembrane activator and CAML interactor” or TACI (6) reflecting this series of interactions. TNFRSF13B potentiates signaling by Toll-like family receptors in B cells (7) and in macrophages (8). Accordingly, TNFRSF13B interacts with MyD88, recruits mechanistic target of rapamycin (mTOR), activates mTORC1 and NF-kB (9–11). TNFRSF13B signaling in B cells generates expression of BLIMP-1, a transcription factor that drives differentiation of B cells into long-lived plasma cells (12).

The importance of TNFRSF13B and BLIMP-1 for development of plasma cells and production of much of the Ig in blood was suggested by investigation of genetic basis of hypogammaglobulinemia, i.e., IgG-deficiency, IgM-deficiency, and IgA-deficiency observed in common variable immunodeficiency (CVID) and in selective IgA deficiency (13, 14). Consistent with this phenotype, TNFRSF13B-deficient mice have few plasma cells in secondary lymphoid organs and in the bone marrow and low concentrations of IgM, IgA, and IgG in serum (12). However, *TNFRSF13B* governs more than the machinery for long term-Ig production. Human subjects with CVID have an increased risk of lymphoma and gastro-intestinal cancer (15) and a propensity for development of autoimmunity (16). Mice with deficient tnfrsf13b exhibit pronounced expansion of follicular and germinal center B cells, despite hypogammaglobulinemia, suggesting tnfrsf13B may govern B cell differentiation and T and B cell interactions (12, 17, 18).

Although some functions of TNFRSF13B, such as control of plasma cell differentiation are understood, some puzzling contradictions remain. One contradiction concerns the impact of TNFRSF13B on the B cell response to antigen. TACI appears more or less essential for natural immunity because humans and mice lacking TACI (targeted deletion in mouse; expression of dominant-negative variants in humans) have extremely low levels of IgG, IgM, and IgA in blood (19) and produce little antigen specific antibodies after exposure to antigen or foreign organisms (20–22). However, most people with dominant negative TACI variants do not manifest immunodeficiency (23) and TACI knockout mice and mice expressing dominant negative TACI variants corresponding to those in humans mount proficient antibody responses and antibody-mediated defenses against pathogenic bacteria (17). Still more puzzling is the relationship between diversity of *TNFRSF13B* genotypes and phenotype. We shall describe recent work that may have begun to clarify apparently disparate aspects of the *TNFRSF13B* phenotype and identify yet unsettled questions we think of importance.

Recent investigations in mice and human subjects have clarified discrepancies concerning the impact of *TNFRSF13B* on B cell responses to antigen. It is now apparent that stimulation of TNFRSF13B is essential for T-independent- but not for T-cell-dependent B cell responses. The requirement for TNFRSF13B (TACI) function for mounting T-independent antibody responses was first shown by von Bulow et al. (24), who found that TACI-KO mice produce less antibodies in response to immunization with pneumococcus. Failure of T cell-independent responses in *tnfrsf13B*-mutant mice was confirmed by Wolf et al. (22) and Mantchev et al. (25), who showed the defective T-independent responses were due to a block in plasma cell differentiation. Tsuji et al. (12) found that defective plasma cell differentiation was due to defective Blimp-1 synthesis. Similarly, Grasset et al. (18) reported that production of gut IgA by T-independent response depends on *tnfrsf13B*. In contrast, human subjects with *TNFRSF13B* mutations, including those with dominant negative phenotype and mice with *tnfrsf13B* deficiency or dominant negative variants respond to antigen when T cell help is provided. Tsuji et al. (12, 17) reported that *tnfrsf13B*-deficient mice are quite proficient in responses to polypeptide antigens associated with enteric organisms (or purified therefrom), generating high affinity antibodies and conferring protection against reinfection. Likewise, Grasset et al. (18) found tnfrsf13B deficient mice quite effectively mount gut IgA responses if T cell help is present.

The differential impact of *TNFRSF13B* on T cell-independent and T-cell-dependent responses may reflect differences in how Blimp-1 is induced. In T cell-independent responses, induction of Blimp-1 depends absolutely on stimulation of TNFRSF13B by BAFF or APRIL (12, 17). In T cell-dependent responses, double strand DNA breaks generated by class-switch recombination (17) and/or engagement of CD40 and IL21/STAT3 (26) signaling can induce Blimp-1 independently of TNFRSF13B signaling. Although TNFRSF13B may not directly impact on Ig isotype class switching, changes of B cell development post-activation by TNFRSF13B mutation, or deletion, that interfere with signaling change the distribution of Ig isotypes in response to stimulation and in the steady state (27).

How TNFRSF13B signaling contributes to the generation and activation of memory B cells remains incompletely understood. The increase in TNFRSF13B expression by memory B cells suggests that TNFRSF13B signaling is important for memory B cell differentiation, survival or function (28). Indeed memory B cells from CVID subjects with monoallelic C104R or A181E mutations fail to activate in response to BCR, TLR7, or TLR9 stimuli (28). Memory B cells of subjects with TNFRSF13B hemizygosity (one null allele and one WT allele) mount a partial response to activation to BCR, TLR7, or TLR9 stimuli suggesting that signaling intensity is important in determining magnitude of response (28). Whether or not the phenotypic manifestations of monoallelic dominant-negative mutations or haplo-deficiency impact memory B cell responses to T-dependent stimuli and in subjects who are healthy is not known.

## *TNFRSF13B* Polymorphisms

The diversity of *TNFRSF13B*, by some measures, equals or exceeds that of genes encoding the major histocompatibility complex (MHC) but the mechanisms contributing to *TNFRSF13B* diversity are less apparent. Diversity of MHC is generally ascribed to the function of housing a vast diversity of microbial peptides for presentation to T cells (29) and while some diversification likely reflects specificity for peptides, some may also reflect non-effector functions such as recognition leading to immune regulation for MHC-class II or controlling NK cells for MHC class I (29).

Yet, *TNFRSF13B* encodes a polypeptide the sole function of which may be recognition of several relatively non-polymorphic agonists, BAFF and APRIL (30). Why then do humans exhibit extreme genetic polymorphism of *TNFRSF13B?* There are 951 *TNFRSF13B* missense and only 383 synonymous mutations reported in compilations of more than 100.000 human genomes (https://useast.ensembl.org/index.html). Only 4 of the *TNFRSF13B* missense alleles are common (freq >0.05). Unlike *TNFRSF13B* variants, nearly 20% of HLA-A variants are found in 5% or more of the population (Genome Aggregation Database, v2.1.1; http://exac.broadinstitute.org). *TNFRSF13B* has a greater number of missense variants than it would be predicted by a sequence-context-based mutational model as reflected by a z score of −1.2, and *TNFRSF13B* has more observed than expected loss of function variants (LoF) (stop gain and splice site variants), which indicate a high tolerance to these types of mutations (pLI = 0.00, where pLI of 1 is the most intolerant) [Genome Aggregation Database, v2.1.1; http://exac.broadinstitute.org; (31)]. Most common variants are phenotypically dominant, either as dominant negatives or causing haplo-insufficiency (32–34) but how exactly expression of mutant alleles results in changes in TNFRSF13B signaling and function is incompletely known (10). In contrast, the number of missense mutations in the *HLA-A* gene is as expected by a sequence-context-based mutational model as (z score of −0.1 according to the Genome Aggregation Database, v2.1.1; http://exac.broadinstitute.org) and the *HLA-A* gene is less tolerant than *TNFRSF13B* to loss of function mutations. Thus, *TNFRSF13B* missense alleles appear to have been selectively retained and recent analysis by the McDonald-Kreitman neutrality index suggests the locus is under strong positive selection (35) in contrast with prior analysis of smaller cohorts (36). This is in contrast to genes encoding HLA which are under moderate purifying pressure (35). Some *TNFRSF13B* polymorphisms are conserved across mammalian species. As an example, mice have 17 missense alleles, 2 non-sense alleles, and 2 splice variants (37).

Adding to TNFRSF13B diversity the receptor is expressed as two isoforms that differ by the presence (long, L) or absence (short, S) of exon 2 that is alternatively spliced following B cell activation (38). The short version of the receptor lacks the cysteine rich domain 1 thought to mediate ligand binding (38). However, absence of the CDR1 domain in the short form does not appear to preclude assembly of the receptor trimer or signaling. Garcia-Carmona et al. (10) showed that TNFRSF13B-S and TNFRSF13B-L assembled receptor complexes composed of one single S or L isoform, or of mixed complexes composed of both S and L isoforms. TNFRSF13B-S requires a lower ligand concentration to signal than TNFRSF13B-L, in part owing to increased ligand binding affinity (10). In contrast, certain mutated isoforms, C104R, A181E, and S194X, produced receptors that had impaired or no signaling (10).

*TNFRSF13B* polymorphisms may be maintained by balancing selection. Balancing selection is thought to occur when multiple alleles (variants) are maintained in the population in an equilibrium, at frequencies more evenly distributed than expected under models of neutral evolution, because selection favors the heterozygote. To measure the likelihood of balancing selection at the *TNFRSF13B* locus we used a statistic, the β index, which detects clusters in close proximity to a site targeted by balancing selection [https://academic.oup.com/mbe/article/34/11/2996/3988103; (39)]. *TNFRSF13B* manifests balancing selection in four regions (two of them between exons 2 and 3, and the other two between exons 4 and 5) (39). Research by Jagoda et al. (40) suggests that certain haplotypes of *TNFRSF13B* may be of Neanderthal in origin and kept in European/Eurasian populations as a result from adaptive introgression. Thus, *TNFRSF13B* variants may have originated by positive selection of archaic ancestry variants, maintained thereafter by balancing selection.

What the selection pressures are that maintain *TNFRSF13B* diversity is not known but our research and the research of others connects these properties to the control of T-independent natural IgA antibody production.

## *TNFRSF13B* and IgA

*TNFRSF13B* promotes IgA synthesis. In support, TNFRSF13B-deficient animals and animals with dominant-negative *TNFRSF13B* alleles are IgA deficient (12, 17, 20) and; selective IgA deficiency in humans is often associated with mutant *TNFRSF13B* alleles (41). We hypothesize that *TNFRSF13B* polymorphisms may in part be driven by the receptor impact on secretory IgA (sIgA).

First discovered in 1953, IgA is by some measures the most abundant Ig in the body (42, 43). IgA exists as a monomer in circulation and as a dimer in lumina of the respiratory, intestinal, and genito-urinary system. Secretory IgA makes 2/3 of all IgA produced in the body (44). An IgA monomer is, like other Ig isotypes, a tetramer composed of two identical heavy chains and two identical light chains united by covalent and non-covalent bonds. Each monomer contains two Fab domains and one Fc region that includes C alpha 2 and C alpha 3 exons (44). Humans, express IgA1 and IgA2, the later encoded by several distinct alleles. IgA1 and IgA2 differ on the hinge region that links constant domain alpha 1 to the constant domain alpha 2 of the heavy chain, longer in IgA1 and shorter on IgA2. The significance of the longer hinge region for the function of IgA1 is not completely known but modeling suggests that the longer hinge region may afford greater flexibility to the variable region relative to the Fc region at the cost of increased sensitivity to proteolysis (44).

The secreted form of IgA is a complex comprised of an IgA dimer linked by the joining (J) chain and a secretory component, a fragment of the polymeric Ig receptor (PIgR). Secretion and dimerization of IgA is made possible by two important adaptations. In one, IgA has an 18 aa tail piece at the C-terminus, highly homologous to another found at the C-terminus of IgM. The tail piece allows IgA (and IgM) polymerization because it binds to the J chain through 2 cysteine disulfide bonds (45, 46). The J chain is a 137 amino-acid polypeptide and it is bound to IgA before secretion. The second adaptation is the development of a highly specific IgA transport system across the epithelium and into secretions. The transport of IgA depends on the polymeric Ig receptor (pIgR) that binds only polymeric Ig (IgA or IgM) and is expressed on the basolateral surface of epithelial cells that line mucosal surfaces of the gut, lung biliary tract, and lacrimal glands. The pIgR has a 620 aa extra-cellular domain, a 23 aa transmembrane domain and a 103 aa cytoplasmic domain (47). Transcytosis of IgA from the submucosal region to the lumen requires binding of dimeric IgA C alpha 3 to the pIgR at the basolateral side of epithelial cells followed by internalization and transport to the apical surface of the cell. IgA is released from its attachment by cleavage of the pIgR ectodomain (secretory component) at the apical surface. IgA, covalently bound to the secretory component is released on to the mucosa, forming sIgA. Bacterial and viral products induce heightened pIgR synthesis by epithelial cells and hence increase transcytosis of IgA (47). The secretory component confers resistance to proteolysis and anti-microbial functions. In addition to binding to IgA, the secretory component exists as a free protein and as such it binds directly to a variety of microbes inhibiting adhesion to epithelial cells, binds to mucus mediating immune exclusion of antigens and pathogens, retains IL-8 and in this way, inhibits neutrophil chemotaxis (48).

IgA complexed with J chain and the secretory component is heavily glycosylated and glycosylation is necessary for many of non-specific, variable region-independent IgA functions (49). IgA complexes are modified by N-linked glycosylation at Asn (N) residues and by O-linked sugars. Glycosylation of the secretory component protects IgA from degradation (50) and mediates some IgA immune functions. Glycans enable secretory component binding to adhesins and lectins, to a wide range of bacteria and to toxins [(48), and references there-in]. Glycosylation of the secretory component determines the distribution of IgA to specific areas of the epithelium (51) and the secretory component is needed with IgA to neutralize rotavirus (52). Furthermore, secretory component free or complexed to IgA, binds to the lectin binding domain of Mac-1 (CR3, CD11b/CD18) enhancing IgA receptor Fc alpha signaling and augmenting phagocytosis and inflammation [reviewed in (53)].

J chain glycosylation is necessary for dimerization of IgA (54) and for maintaining the correct conformation to facilitate interaction with pIgR (55, 56). Serum IgA and secretory IgA are differentially glycosylated presumably reflecting differences in the glycosylation machinery in plasma cells of the spleen and bone marrow vis a vis plasma cells in the mucosae (48).

The heavy-chain N-linked glycans help maintain the correct IgA conformation and assist with dimer formation and secretion (57, 58). Although the glycan composition of IgA HCs activate the lectin complement activation pathway by binding mannose binding lectin (MBL), the glycans are shielded by the secretory component at physiologic pH and only become accessible for binding at low pH and perhaps upon engagement with bacterial adhesins which pull the secretory component away from the IgA (48). Once uncovered, IgA HC glycans may also directly bind mannose receptors on phagocytic cells promoting internalization and antigen presentation (59). Secretory IgA HCs are also extensively modified by O-linked glycans which form highly complex structures that in addition to stabilizing the hinge region also interact with bacterial adhesins (60). Glycosylation-dependent IgA functions are likely due to low affinity interactions and therefore critically dependent on IgA concentration. Although there is no evidence in support of TNFRSF13B direct influence on IgA glycosylation, by controlling IgA secretion TNFRSF13B is likely to impact more on the non-specific IgA functions than on the functions of IgA that depend on high affinity interactions between mutated V regions and their targets.

## Discussion

One might expect that a cytokine receptor such TNFRSF13B would exert straight forward functions that promote host defense and/or immune regulation and accordingly polymorphism should be rare and harmful. Yet, TNFRSF13B is as diverse as MHC. Why?

Although the story is yet incomplete, the complexity of TNFRSF13B gene and protein functions has been coming more fully into focus. Although TNFRSF13B does not bind antigen it controls response of B cells to T-independent and to T-dependent antigens differently. TNFRSF13B promotes T-independent antibody production in part by facilitating differentiation of plasma cells. In contrast, TNFRSF13B-deficiency does not inhibit and in some cases enhances IgG or IgA responses to T-dependent antigens.

Most sIgA results from T-independent B cell responses. IgA exerts protective functions by helping to eliminate pathogens directly and/or through maintaining microbial homeostasis and these functions are in a great extent independent of IgA specificity (61–63). The equilibrium established between IgA, microbes and pathogens results from anti-microbial functions of IgA and microbial adaptations to IgA (49, 64). In one example of a remarkable microbial adaptation, Nakajima et al. (49) showed that highly glycosylated IgA of an irrelevant specificity (specific to ovalbumin) bound to a human symbiont *Bacteroides thetaiotaomicron* (*B. theta*) changing gene expression. IgA induced transcription of *B. theta* polysaccharide utilization loci (65) changing polysaccharide utilization and in this way conferring competitive advantage (49). As another example, *Bacteroides fragilis* capsule induces polysaccharide specific IgA which in turn, increases its adherence to intestinal epithelial cells by binding to mucus as well as to the capsule polysaccharides (62). In these examples, commensal bacteria co-opt IgA to colonize the gut.

**Figure 1:**
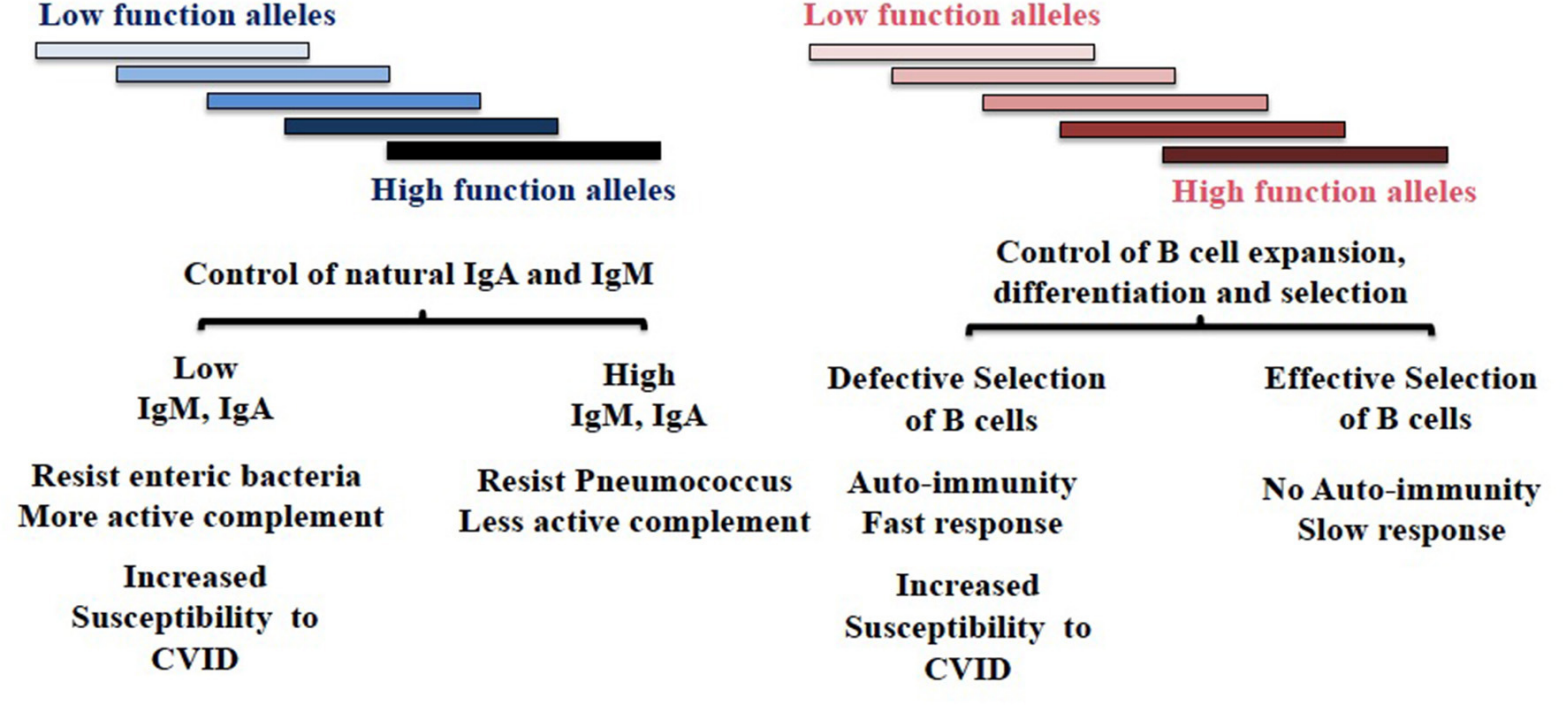
Diversity of *TNFRSF13B* in host response to environmental challenges. *TNFRSF13B* encodes a receptor (TACI) through to support the maturation of B cells, yet the gene is among the most polymorphic in the genome and polymorphism is maintained across mammalian species. The authors speculate that polymorphism of *TNFRSF13B* reflects balancing selection—balancing benefits and disadvantages of signals the receptor delivers but also manifold intermediate functions the protein and its neighbors in cell membrane confer. The figure depicts two hypothetically distinct regions of the protein that confer phenotypically distinct properties. The balancing of complex sets of phenotypic properties that presumably impact all mammals could underlie extraordinary diversity of the gene across species. CVID, Common Variable Immunodeficiency.

We hypothesize *TNFRSF13B* polymorphisms are maintained at least in part as the result of microbial adaptation to IgA (Figure 1). We offer the following reasoning in support of the hypothesis.

In a recent report Grasset et al. (18) showed that TNFRSF13B is necessary for T-independent IgA responses to commensal bacteria. These responses include secretion of polyreactive IgA and represent the majority of secreted IgA. In contrast, T cell-dependent IgA responses occur independently of TNFRSF13B. Thus, varying TNFRSF13B function changes the ratio between highly-specific and polyreactive or non-specific IgA responses which, in turn, may counteract pathogen adaptation to IgA and facilitate specific elimination. In support, Grasset et al. (18) showed that *tnfrsf13b-KO* mice generated mutated IgA that specifically targeted a restricted subset of microbes. In an earlier publication, Tsuji et al. (17) showed that *tnfrsf13b-KO* produced highly mutated antibodies that rapidly cleared *C. rodentium*. Whether changes of the properties and amount of sIgA and/or varying the proportion of mixed polyreactive and specific mono-reactive Ig in individuals or mice expressing *TNFRSF13B* polymorphisms is protective awaits investigation.

For all the benefits of IgA, selective IgA deficiency is often asymptomatic (41). *TNFRSF13B* polymorphisms are associated with IgA deficiency (41), the most common immune-deficiency (with frequencies varying between 1:143 in the Arabian peninsula to 1 in 500 Caucasian individuals) (66, 67). The limited morbidity of IgA deficiency, suggests as one possibility that benefits conferred by TNFRSF13B variants may outweigh the detrimental impact of decreased IgA in the gut. Because *tnfrsf13b* mutants maintain the ability to make “adaptive” IgA it is possible that protective functions of IgA are maintained or even enhanced. In accord, IgA-deficient patients were found to have enhanced adaptive antibody responses to pneumococcal vaccination (68). Perhaps it is this type of response that explains the mild phenotype of many individuals with IgA deficiency and the maintenance of the extreme polymorphism at the *TNFRSF13B* locus.

## Data Availability Statement

The original contributions presented in the study are included in the article/supplementary material, further inquiries can be directed to the corresponding author/s.

## Ethics Statement

Animal studies and human subject research that inspired some of the hypotheses in the manuscript were reviewed and approved by the Institutional Animal Care & Use Committee (IACUC) or by the Institutional Review Board (IRB) at the University of Michigan.

## Author Contributions

MC and JP wrote the manuscript. MC analyzed data. All authors contributed to the article and approved the submitted version.

## Conflict of Interest

The authors declare that the research was conducted in the absence of any commercial or financial relationships that could be construed as a potential conflict of interest.
